# Promises Past and Future - Gene therapy and the actualisation of future expectations

**DOI:** 10.1016/j.socscimed.2025.118794

**Published:** 2025-11-14

**Authors:** Eva Hilberg, Tineke Kleinhout-Vliek, Aleksandra Stelmach, Paul Martin

**Affiliations:** iHuman Institute, School of Sociological Studies, Politics and International Relations, University of Sheffield; Sheffield, UK; School of Business, University College Dublin; Dublin, Republic of Ireland; Department of Management, University of Exeter; Exeter, UK; iHuman Institute, School of Sociological Studies, Politics and International Relations, University of Sheffield; Sheffield, UK

## Abstract

The advent of gene therapies such as *Zolgensma, Libmeldy*, and *Luxturna* has given rise to new treatment options for several rare conditions, drastically changing the expectations of affected patients. It has also significantly influenced hopes of medical treatment in general, with an emerging vision of widespread targeted personalised treatment of increasingly segmented conditions. Looking at a newspaper sample from the latest wave of developments in the field (from 01/01/2020 until 30/04/2023), our analysis of current media coverage however finds that this narrative of paradigmatic change operates mostly without regard to the present and its challenges, such as the prohibitive price tag of these treatments and unresolved questions about their accessibility and long-term effects. Drawing on expectations raised in the context of the completion of the Human Genome Project in 2000, the article compares these to current hopes of gene therapy’s safety and effectiveness; profitability; and accessibility. Areas of tension are then interpreted as a guide to an emerging ‘real-life’ understanding of gene therapy’s promise, which is, however, mostly visible in discussions of problems with gene therapy’s accessibility. Similarly marginalised issues include for instance assumptions of effectiveness that do not acknowledge the long-term uncertainty of treatment outcomes; and assumptions of profitability that run counter to real-life examples of business failure. As a revolutionary future becomes reality for some patients, such questions are becoming harder to ignore – but are crucially often omitted from discussion about projected change.

## I Introduction

The development of gene therapies has been a field of promise from the beginning. Hotly anticipated, the vision of the future of these extremely targeted treatments was revolutionary, and a key part of the promised “new era of genetic medicine.” (The White House 2000a). Several decades on, with the advent of gene therapies such as *Zolgensma, Libmeldy*, and *Luxturna*, this widely-held expectation of medical revolution appears to finally become reality. As new treatment options for a number of rare diseases begin to give a face to the abstract notion of genetic medicine, these expectations are themselves also undergoing a revolution. This article takes a closer look at the current state of gene therapy’s original promises, and the way in which they are re-worked in current perceptions. As real treatments are being taken up by health systems more broadly, this moment shows how a variety of stakeholders are re-evaluating their expectations in terms of their experience – and are discussing their current future hopes in different types of media. The following media analysis shows how some of the original future promises continue to influence developments even in the face of contrary experience, while others are becoming more difficult to maintain. As different health systems begin to grapple with this new challenge, the debate around the promise of emerging gene therapies is only just beginning. We argue that charting the increasingly defined promise of gene therapies at this juncture thus can reveal the structure and the structuring effects of what we currently understand as gene therapies; revealing public, scientific, regulatory, and patients’ expectations while also showing the shortcomings of the present debate.

There have been many different promises about the effects of gene therapy, some go as far as saying “[g]enomics *must* be analyzed in terms of the promise, because promising is an ineradicable feature of genomics” (Fortun 2008, 10, emphasis in original; see also Brown’s work on expectations and genomics, in Brown 2003). Taking the seminal press conference announcing the completion of the Human Genome Project at the White House on 26.6.2000 as a point of departure, the article contrasts these erstwhile expectations with the current state of promises regarding gene therapy’s safety, profitability, effectiveness, and accessibility – topics that are still at the core of debates. Instead of a gradual re-evaluation of expectations (Brown and Michael 2003), our analysis finds an overall enduringly positive assessment of gene therapy’s promise, even in the face of contrary real-life experience. However, different issues are not being afforded the same amount of exposure and discussion within this coverage, and most issues are being discussed in isolation of each other. Our analysis shows how different themes are frequently revisiting the more enthusiastic hopes surrounding individual gene therapies. We understand this accumulation of sensationalist promise interspersed with critical voices as the collision of a regime of hope and an emerging regime of truth (Moreira and Palladino 2005), which is also part of a process of increasing definition of gene therapy in public perception. Truth, in this context, represents the emerging real-life experience that should act as a check on the most rampant expectations – which, however, thus far has not made too many inroads on the exceptionally high hopes surrounding the emergence of gene therapy.

After surveying the original set of promises made at the beginning of the age of popular genetic medicine - the completion of a rough draft of the Human Genome announced at a White House conference in 2000 – this article sets out different ways of studying promise and expectations. Using the original set of promises emerging around the year 2000, we then go on to review different themes and contestations arising around each area of promise: 1) Promises regarding Safety and Effectiveness; 2) Promises regarding Profitability; and 3) Promises regarding Accessibility. Taking a closer look especially at critical pieces – as these are rare – we then discuss overlaps and oversights between these areas. These areas of tension are then interpreted as a guide to an emerging ‘real-life’ understanding of gene therapy’s promise, which is, however, mostly visible in discussions of accessibility. This discussion finds several blind spots, such as for instance assumptions of effectiveness that do not acknowledge the long term uncertainty of treatment outcomes; assumptions of profitability that run counter to real-life examples of business failure; and the conflicted role of patients who are both contributing their experience as the recipients of this new medicine, and also are having to gain attention and access to treatments. We argue that the sheer amount of positive accounts even in the face of uncertain or disappointing treatments can be seen as part of the constant (re)making of a broader imaginary of a revolutionary personalised and genetic or genomic medicine, despite relatively slow progress overall.

## II Promises past and future

Expectations around gene therapies have always been high and were part of the genomic project from the beginning. One of the key moments of its development in public perception was undoubtedly the widely covered White House press conference on 26 June 2000, announcing the completion of the Human Genome Project after a race between two different research institutes to complete a rough working copy of the human genome (the first full human genome was sequenced on 14.4.2003). While the concept of gene therapy predates this announcement – it emerged in the 1970s (Friedmann and Roblin 1972; see also Dunbar et al. 2018) – this event brought together US president Bill Clinton, UK Prime Minister Tony Blair, Dr Francis Collins (Director of the National Human Genome Research Institute), and Dr Craig Venter (President and Chief Scientific Officer of the Celera Genomics Corporation), and purposefully created a high-profile moment of scientific triumph with global reach at the turn of the century. President Bill Clinton set the scene and spelled out some of the expectations surrounding this event: “Today’s announcement represents the starting point for a new era of genetic medicine.” (The White House 2000a) The implications of this shift would be of a magnitude that meant “it is now conceivable that our children’s children will know the term cancer only as a constellation of stars” (The White House 2000b).

But it took a long time (and several other revolutions) for this type of medicine to become a tentative reality – over fifty years, in fact, from the first notion of “genetic modification by exogenous DNA” to its realisation (Dunbar et al. 2018, 175). Gene therapy is defined by the FDA as “a technique that modifies a person's genes to treat or cure disease.” (FDA 2018). By now, a number of gene therapies are available to patients in several countries, for instance a treatment for particular types of retinal dystrophy (*Luxturna*) and also, most prominently, a gene therapy for a specific type of spinal muscular atrophy (*Zolgensma*). Starting from the first US-approved gene therapy in 2017 for acute lymphoblastic leukemia (*Kymriah*), by 2023, the tally stood somewhere between “22 approved in vivo and ex vivo products” (Shchaslyvyi et al. 2023) and “more than 100 different approved gene, cell, and RNA therapies throughout the world, with over 3,700 more in clinical and preclinical development.” (Chancellor et al. 2023, 3376) This number includes different types of gene therapy, mainly divided into *in vivo* and *ex vivo* therapies according to their route of delivery. This type of therapy works on the basis of either switching off a faulty gene sequence, or inserting, or even replacing, this faulty sequence with a corrected version. In *in vivo* interventions, a viral vector is used as a “shuttle” that delivers the correct sequence to the target cell type (Pfeifer and Verma 2001). In *ex vivo* therapies, patient cells are modified outside the body and then reintroduced to take effect (Gowing et al. 2017, 99). New technologies emerge constantly, such as gene editing, which is “the introduction of a predetermined sequence change to the chromosomal DNA of a cellular genome” by means of technologies such as CRISPR (clustered regularly interspaced short palindromic repeats)-Cas (CRISPR-associated) nucleases systems (Xu and Li 2020, 2401). After over fifty years of development, these types of therapies are finally beginning to deliver on the original promise “that a durable and possibly curative clinical benefit would be achieved by a single treatment” (Dunbar et al. 2018).

The promises of gene therapies are often seen as virtually limitless and encompass several elements. A Pfizer explainer of gene therapies states: “Gene therapy could enable patients to live without the need for ongoing treatments or the burden of daily disease management” (Pfizer 2025). This has enormous potential, especially in the field of rare (genetic) conditions, as stressed by both Pfizer and also by a meta-review of the opinions of 1430 researchers working on rare disease: “Overall, they expect genetic therapies to be the standard of care for rare genetic diseases before 2036 and to cure them after this period.” (Braga et al. 2022, 12) A 2005 review evaluates progress towards this “Twenty-first century medicine”: “Rarely comes along a modality of medicine that has the prospect of such widespread and profound influence on human health. The young field of gene therapy promises major medical progress toward the cure of a broad spectrum of human diseases, ranging from immunological disorders to heart disease and cancer.” (Verma and Weitzman 2005) All of these brief statements revolve around the notion of a ‘cure’ – which may, however, not be as straightforward or long-lasting as commonly assumed. In fact, first experiences with the therapy for haemophilia are shown to be “effective, at least for several years, but the exact effect may be unpredictable […]” (Chou et al. 2023). This disparity between expected miracle cure and real-life complexity shows that the original promise of gene therapy will have to be critically re-evaluated over the coming years.

The wide range of promises surrounding the emergence of gene therapies goes back to the early days of genomic research that is the foundation of this type of targeted treatment. The White House announcement of the completion of a working draft of the Human Genome Project contained a number of these promises, as it stated that “scientists will be able to use the working draft of the human genome to: [….] precisely diagnose disease and ensure the most effective treatment is used” and that “[d]rug design guided by an understanding of how genes work and knowledge of exactly what happens at the molecular level to cause disease, will lead to more effective therapies” (The White House 2000a). Increased accuracy of treatment would mean fewer side-effects, and an increase in treatment safety: “patients with some forms of leukemia and breast cancer already are being treated in clinical trials with sophisticated new drugs that precisely target the faulty genes and cancer cells, with little or no risk to healthy cells.” (The White House 2000b) The influence of this new medicine would be pervasive: “Genome science will have a real impact on all our lives — and even more, on the lives of our children. It will revolutionize the diagnosis, prevention and treatment of most, if not all, human diseases.” (The White House 2000b) At the time before this announcement, this new molecular medicine was also already expected to be vastly profitable, as exclusive rights to gene sequences would enable companies to “control the next era of health care” (Fisher 1999, 1).

The revolution thus promised to be not only safe and effective, but also profitable and accessible. The next twenty-five years brought some hope but also disappointments, such as failed trials (Sibbald 2001), the demise of companies (see below), and the increasingly exorbitant price of treatments. Twenty-five years after the announcement, these real-life experiences of gene therapy provide an opportunity to re-visit original promises, in order to see in how far expectations have undergone change in the cold light of day. The following sections undertake a thematic analysis of the debate around gene therapies and find that instead of an increasing loss of expectations in the face of reality, there is an extraordinary resilience of expectations around gene therapies.

## III The study of future expectations

The pivotal influence of expectations on industrial and regulatory development has already been studied critically in a variety of ways. The sociology of expectations focuses especially on the interactions between anticipated technological developments and the emergence of particular regulatory regimes that seek to promote this development (van Lente 2012; Brown 2003). Van Lente conceptualised these anticipations as “forceful futures”, which “mobilise attention, guide efforts and legitimate actions” (van Lente 2000, 43). Others have focused on different regimes of hope and their connection to creations of value (Novas 2006), for instance in the case of stem cell technology (Martin, Brown and Turner 2008), the development of neurotechnology as a new industrial sector (Martin 2015), and also the shaping of pharmacogenetics (Hedgecoe and Martin 2003). There has also been study of the sociology of *low* expectations, which can work as a process of simplification in policy-making (Broer and Pickersgill 2015), as a tool of managing risk contained in “futures to be avoided” (Tutton 2011), and a recalibration of expectations in a clinical setting to enrol patients in an “innovation alliance” (Gardner et al. 2015). To this literature we aim to add an account of the actualisation of expectations, seen as the process of imaginaries and realities colliding – or in the way in which particular “regimes of hope” encounter a check in emergent real-life “regimes of truth” (Moreira and Palladino 2005). This moment should trigger a re-assessment of expectations – but can, as in the case of gene therapy, also show a surprising resilience of expectations in the face of contrary evidence.

Our analysis looks more closely at the development of future expectations over time, as for instance proposed by Brown and Michael (2003). While their study focuses on the development of expectations in the light of real-life disappointment, ours deals with the moment of actualisation in which a wildly optimistic, general hope gives way to a more specific and detailed regime of expectation – but in which also, as our study shows, these two elements still coexist in an increasingly uneasy relationship. Literature has so far focused on the creation of future expectations, and conflict between different visions (Borup et al. 2006; van Lente 2012; Konrad 2006; Hausstein and Lösch 2020; Tavory and Eliasoph 2013; Suckert 2022). A longer-term perspective on expectations has been explored in the context of non-medical technologies, such as for instance photovoltaic technology (Kriechbaum et al. 2018), fuel cells (Budde and Konrad 2019), smart meters (Hielscher and Kivimaa 2019), alternative fuel vehicles (Melton et al. 2016), and biomass gasification (Kirkels 2016). These assessments point to a phase of disappointment (or a “trough of disillusionment”) after a hype runs its course – in this phase, expectations adjust in a more sedate process of increasing “enlightenment” (original terminology from Gartner Consultancy Group, quoted after Kirkels 2016, 85). This process of enlightenment has not taken place in public perceptions of gene therapy, where instead, the first wave of hype-disappointment seems to have given way to a second wave of strong interest. Our analysis shows that expectations in the public domain are extraordinarily resilient, even in the face of real-life experiences that should challenge them. The example of gene therapy thus differs from the infrastructural technologies mentioned above (for instance constantly changing alternative fuel vehicle technologies in Melton et al. 2016) as the technology is now becoming widely adopted, and yet does not produce more measured coverage. Instead of enlightenment, this could potentially be indicative of a second ‘hype’ that is so far impervious to challenges.

**Figure 1:**
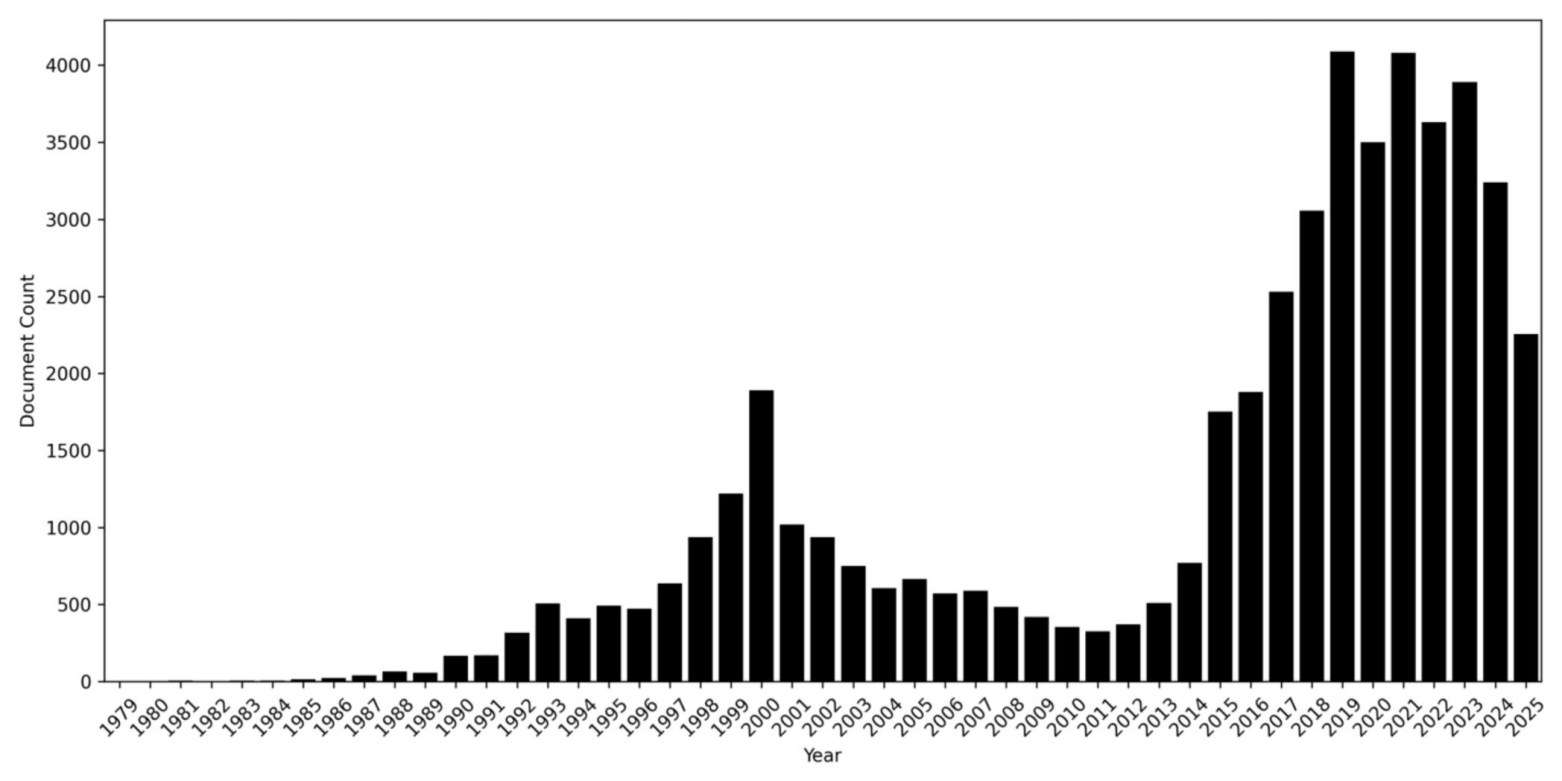
Reporting on gene therapy in top English-language sources across the world according to Factiva 1979-2025.

**Figure 2:**
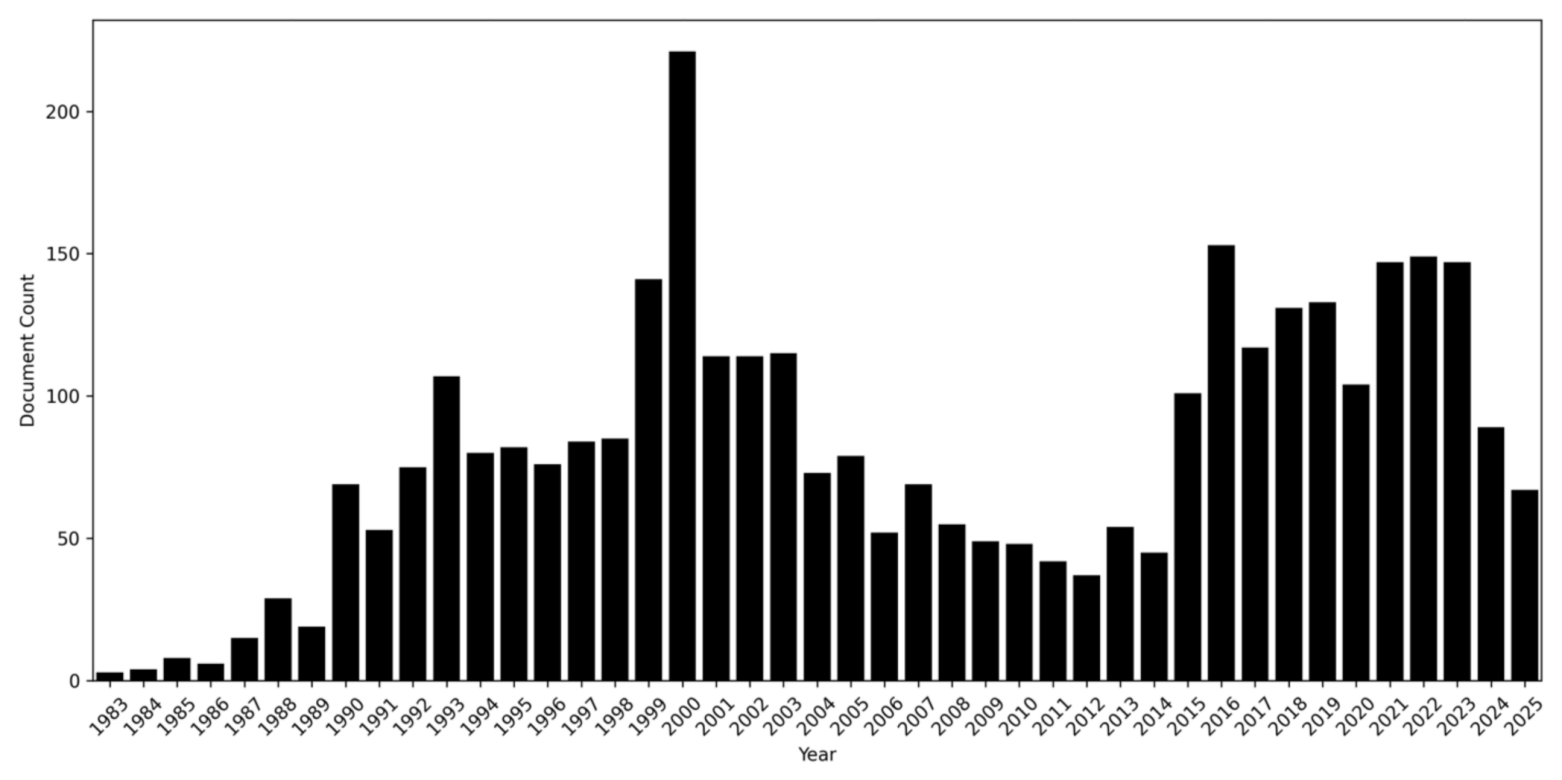
Reporting on gene therapy in sources included in sample according to Factiva 1983-2025.

We argue that the case of gene therapy provides a unique opportunity of studying a well-established “ecology of interacting futures” (or “economy of promises”, Felt and Wynne 2007; also Brown and Rappert 2000) undergoing the realisation of its core expectations. In our sample, this moment gives rise to media coverage that exceeds the initial wave of interest around the time of the Human Genome Project (see Figure 1; the total amount of media publications has however also increased overall in this period making it difficult to judge a relative increase – in fact, Figure 2 shows a more balanced amount of publications within both time frames). Different experiences of actual treatments and the emergence of specific new therapies were covered closely in a wide variety of media outlets, ranging from relatively high-brow newspapers to more sensationalist press. These less formal settings outside of the scientific and industry canon are places in which public expectations are formed and can be studied (see for instance Kriechbaum et al. 2018; Petersen et al. 2005). Here, we trace the actualisation of public expectations regarding the main expectations of gene therapy, seeking to find incremental adjustments of these expectations within current debate. We look at these developments as a regime of hope undergoing a process of actualisation, understanding current debate as a social “process of collective definition” (Hilgartner and Bosk 1988, 53). This perspective can draw attention to the conflicts contained in this process, and their often imperfect result in which real-life concerns are overshadowed by continued high expectations.

## IV Methods

In this study we conduct a thematic analysis of media coverage of the term “gene therapy” in selected UK and US media outlets. Taking the original hopes surrounding gene therapy as a point of departure, the analysis traces its problematisation in contemporary media coverage within a selection of UK and US newspapers from 01/01/2020 until 30/04/2023. The aim of the analysis is: 1) to discover which future promises are being represented in these reports; 2) which issues are commonly understood to be at stake in the field of gene therapy; and 3) how these future visions and emerging real-life issues interact in a process of increasing definition of gene therapy’s promise in public perception.

Our sample focuses on reporting from the beginning of the current decade to capture the emergence of actual gene therapies. This makes a deliberate decision to produce a relatively focused sample, and excludes the first wave of hype around the year 2000, at the time of the completion of the Human Genome project. As figures 1 and 2 show, instead of a slow adjustment to reality, the current wave of reporting within our sample rather indicates a second phase of interest.

We used the media database *Factiva* to search for articles with the term ‘gene therapy’ in UK and US media, two countries with a significant industry in this area. In each country we chose three newspapers with the largest volume of published articles on gene therapy. In the UK, we selected *The Times*, the *Daily Mail*, and *The Independent*; and in the US we selected *The New York Times*, the *Boston Globe*, and *USA Today*. The search returned a sample of 447 articles (194 from the UK and 253 from the US). We excluded duplicates as well as articles where gene therapies were mentioned in an entirely different context, such as the COVID-19 vaccine debate. The final sample consisted of 176 articles (69 articles from the UK press and 107 from the US press).

**Figure 3:**
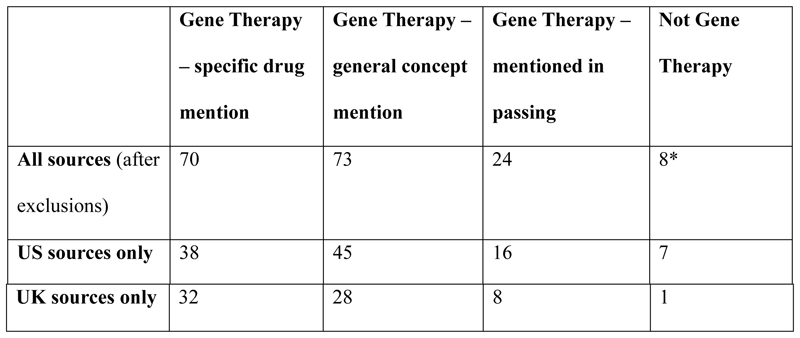
Types of gene therapy coverage in sample. *Of those articles that are not about gene therapy, 4 are about monoclonal antibodies, 2 about vaccines, one about a chemical drug, and one a targeted therapy medication.

The US sample is bigger than the UK one – as generally speaking media coverage of gene therapy is largest in the US. But it also turned out to be the more varied sample in terms of definitions of gene therapy – these, as Figure 3 shows, could include treatments that are not in fact gene therapy at all according to the definition used above. Across the entire sample, of those articles that were not about gene therapy, 4 were about monoclonal antibodies, 2 about vaccines, one about a chemical drug, and one a targeted therapy medication. While the definition of gene therapy is slowly shifting to accommodate new technologies such as gene editing, the public perception of gene therapy thus seems to be expansive and not as clearly defined as the scientific understanding of this term. This highlights the imprecision of public media coverage, but also arguably the power of the gene therapy imaginary, which is emerging as a key point of reference for advanced therapy medicinal products more generally. The focus of our discussion is on expectations of gene therapy in public perception, and our discussion below marks clearly where a statement refers to a treatment that is not strictly speaking gene therapy – however, the points made with regards to the impact of cost of advanced treatment apply nonetheless.

Two of the authors read the sample twice and met regularly online to analyse the texts and develop and discuss the thematic issues covered in these. The following discusses these themes in reference to each original promise – 1) Promises regarding Safety and Effectiveness; 2) Promises regarding Profitability; and 3) Promises regarding Accessibility.

## V Stories of Promise – Gene therapy in the media

Media coverage of gene therapy was consistently positive over the period under consideration. Each theme (Safety and Effectiveness; Profitability; Accessibility) was discussed predominantly in a positive light – indeed, truly critical voices were remarkably hard to find. However, the issues at the heart of the adoption of gene therapy were often visible in the background, and some pieces were engaging more directly with potential discrepancies between expectations and reality. These are the stories that we focus on most closely, to see how these different expectations are being reworked in the light of real-life experience. These more complex news items are, as argued above, part of a process of actualisation that redefines the contemporary understanding of the promise of gene therapies. However, we want to stress that these stories are only a fraction of the generally positive coverage. This imperviousness emerges as one of the core features of gene therapy reporting.

### A Promises regarding Safety and Effectiveness

Across our sample, gene therapies consistently feature as one of the most extraordinary achievements of contemporary science: they are hailed as cutting-edge technologies bringing hope and cures to people afflicted with many serious conditions. Their effectiveness was often described in glowing terms, whereas challenges surrounding safety and effectiveness were understood as momentary roadblocks. In the words of Dr Francis Collins, who led the Human Genome Project and was now the director of the National Institutes of Health in the USA, gene therapies were “an exhilarating success story for those of us who have waited and hoped for this day” (*The New York Times* 12/01/2020). Similar to many other emerging technologies covered in the media, discussions of gene therapies drew on the conventional language of scientific breakthroughs and revolutions. For example, gene therapies were described as “pioneering treatments”, and exciting innovations “at the absolute cutting edge of scientific understanding” (*Daily Mail* 25/10/2022). Some of them did, however, acknowledge the delayed success of gene therapies – but emphasised that these challenges had now been overcome, linking these to the promises to the Human Genome Project:

Back in 2000 Bill Clinton announced the first draft of the human genome, or as he called it, a map. (…) In the decades since, many have entered a trough of disillusionment about the promise of genetics. In his speech Clinton promised us personalised medicine, personalised disease risk, precise diagnostics, molecular medicine. Where are they? But, quietly, science pulled itself from that trough. Not so much by doing new things but by doing old things better. Molecular scissors called CRISPR came along that could edit DNA. We could edit DNA before, but they made it easy. (…) We know it because, like jet engines and computers before, genetics is becoming mundane. (*The Times* 31/05/2021)

Gene therapies were thus described as a quiet scientific revolution of sorts, made possible by other technological advances, such as gene editing, along the way. The results were “easy”, or even “mundane” – implying a reliability and effectiveness that is no longer out of the ordinary. Challenges, or the “trough” of stalled development, were occasionally mentioned, and pertained to failed trials, leading in some cases to deaths of patients. For example, an article in The *Boston Globe* recounted the historical “disaster” of one clinical trial of gene therapy from over two decades ago in which “a patient (…) had an immune response that led to organ failure and death. As a result, the entire field, which had been on the cusp of developing treatments, was effectively frozen” (*The Boston Globe* 06/06/2020). Concerns about safety and effectiveness were thus a reason for the delay, but – by implication – were now over.

In fact, many articles emphasised that, despite these setbacks, the field was now growing. Especially the COVID-19 pandemic had prompted “a Manhattan Project-sized boost” for genetics, and meant that this science had “quietly saved the world” (*The Times* 31/05/2021). This was because the development vaccines for the novel coronavirus relied on the same revolutionary approaches as those used to create gene therapies. The pandemic thus renewed an interest in the potential of genetics to develop treatments. As one article in *US Today* suggested, after the completion of “Operation Warp Speed” regarding the rapid development of COVID-19 vaccines, the minds of scientists and policymakers were now set on gene therapies, the next big thing in science (*USA Today* 23/06/2021). This was akin to an “exponential growth phase”:

Within the next few years, experts say, gene therapies could soon be available for conditions never effectively treatable before, such as sickle cell disease, Huntington's, ALS, Parkinson's, some forms of heart disease and a host of very rare diseases. "The exponential growth phase" of gene therapy has arrived’ (*USA Today* 27/04/2021).

The safety and effectiveness of new gene therapies were central to these descriptions. Articles often described cutting-edge techniques, including new viral vectors delivering genes to cells and tissues, as well as new types of genetic manipulations such as CRISPR, base-editing and epigenetic editing techniques. Evoking President Clinton’s vision of future treatments, these were pronounced to be more precise, and, potentially, safer than techniques used in previous studies. Scientists were now using a “new, ultra-precise gene-editing” that could get “safely” straight into the desired tissue (*The Independent* 29/07/2022). CRISPR was framed in the press as a “precise genetic tool” which could be used not to cut out a gene, but to block its effect, which would be safer and even reversible. (*The Times* 06/01/2020). The new editing tools thus appeared to provide better control over the genome: “We really need ways to correct genes, not just disrupt them. And that's where base editing comes in. To be able to take control of our genomes, to me, is one of the most human things we can do.” (*The Boston Globe* 12/07/2022). The new gene therapies were also depicted as relatively easy to use, and often consisting of a “single-shot jab” (*Daily Mail* 03/08/2021) or “one-and-done treatments” (*The Boston Globe* 08/06/2022). “A single treatment”, one article claimed, “could protect males with haemophilia from dangerous bleeding for years” (*The Times* 18/11/2021). Closely mirroring the original promises around safety and effectiveness, gene therapies were thus described as successful treatments able to stop diseases from progressing or even curing them once and for all.

### B Promises regarding Profitability

The development of gene therapies was continually framed in the press as a key opportunity for investment in biotechnology and pharmaceutical companies, something that was covered in great detail in business and finance sections of the analysed newspapers. Gene therapies were seen as key areas for “rebuilding the economy” and investing in them could provide “a good return to pension savers” (*Daily Mail* 06/08/2020). The media closely followed activities of companies developing gene therapies. For example, a venture capital firm with gene therapies in its portfolio was lauded in the press for its “phenomenally high rate of return”. This was because

At the end of 2019, a dollar invested in one of Flagship's funds would have been worth about $9. That return multiple — a measure that investors use to describe a fund's relative value — was better than the return multiple recorded by any other life sciences venture capital fund since 2000. (*The Boston Globe* 23/02/2021)

At the same time, setbacks such as technical difficulties, problems with regulatory approval, disappointing trials or unexpected side effects of gene therapies in patients were considered as a challenge that could undermine investors’ confidence. The media reported for example on halted clinical trials as patients undergoing experimental gene therapy treatments had developed cancer, or even died from side effects (*The Boston Globe* 04/11/2022). Yet hopes for novel treatments and positive news about actual achievements in developing gene therapies kept the confidence in these technologies afloat. “When you step back”, one article noted, “the scientific advancements in gene and cell therapies achieved in the past 10 years are simply astounding. Certain types of rare, inherited diseases can be cured by replacing malfunctioning genes with ones that are healthy and function normally” (*The Boston Globe* 01/01/2020). Biotech and pharmaceutical industries were developing “world-changing innovations” (*The Times* 27/06/2020), “life-saving gene therapy drugs” (*The Times* 29/01/2022) and even “miracle treatments” (*The Independent* 15/02/2023) that were beginning to change the face of healthcare.

There were significant overlaps between discussions around profitability and discussions around safety and effectiveness, for instance the emphasis on temporary roadblocks that had now been overcome. But this interpretation does not always reflect the whole story, as the example of Bluebird Bio shows. One article reported about the company awaiting the decision of the FDA on their one-time therapy, *Skysona*, will would be sold for $3 million:

“To have reached this moment after a decade of research and development, with many setbacks along the way, is truly remarkable,” [chief executive] Obenshain said. The positive news has helped the company's stock double since its low in June, but it is still down more than 94 percent since its high in 2018. (*The Boston Globe* 17/09/2022)

Despite these good news, Bluebird Bio has since faced financial hardship with several rounds of lay-offs, diminishing stock price, and is currently being sold to new investors. These developments show that assumptions of vast profitability of gene therapies do not necessarily bear out in reality.

The issue of increasingly stratospheric prices for treatments also received attention, especially in reports on successive gene therapies breaking records in terms of their high costs. For example, the media reported on the drug *Zolgensma* developed for treating spinal muscular atrophy (SMA), a genetic condition affecting children, that, at the prices of £1.7 million, was “thought to be the most expensive drug in the world” (*Daily Mail* 15/12/2020). Two years later, the gene therapy *Skysona* for a rare brain disease in children was called “the most expensive drug in the country” at the price of $3 million per treatment (*The Boston Globe* 17/09/2022). In 2023, *Hemgenix*, the gene therapy for hemophilia, was branded once again “the world’s most expensive drug” at the price of $3.5 million per patient (*The Boston Globe* 29/01/2023). Prices for gene therapies were so high, because, as one pharma executive explained:

Not only are the new drugs costly to research and develop but some treatments are for just a few patients with very rare diseases and some, like gene therapy, are used only once rather than over a person's lifetime. The prices of today's remedies reflect all those factors. (*The New York Times* 07/02/2023)

In addition, some argued that gene therapies, often in the form of one-off treatments, could actually save costs. As one article argued:

The one-time treatments carry jaw-dropping price tags. (…) such expensive medicines may actually be cost-effective by curing debilitating diseases that can strain the health care system if treated for years with conventional drugs and hospitalizations. (*The Boston Globe* 11/01/2021)

This calculation works on several assumptions – that these drugs are given only once in a lifetime, and that they are entirely curative and thus need no subsequent therapy with conventional drugs. While there were fears that high prices could prevent patients accessing treatments, there were also hopes that the prices would eventually come down, as some companies were working with insurers to make sure the treatments would be covered once approved (*USA Today* 16/06/2022).

### C Promises regarding universality and accessibility

The promise of the accessibility of treatment virtually for *all* had been part of the original vision for this new era of medicine, as set out above. This promise can still be traced in current media coverage, but it is also increasingly questioned in more critical pieces. In the words of the developers of CRISPR, gene editing tools underpinning gene therapies offered a “profound opportunity to change health care for many people” (*The New York Times* 11/12/2022), and there were hopes that “world-changing innovations are just round the corner” (*The Times* 27/06/2020). However some researchers and healthcare analysts argued that such prospects were unrealistic in the short term, especially given their high costs. The long and expensive process of developing gene therapies, especially for rare genetic conditions, meant that claims about the widespread adoption of precision medicine were – at least for now - largely unrealistic. In the words of one expert, this was simply “not commercially viable” (*The New York Times* 11/12/2022).

The development of Bluebird Bio was seen as a significant case study in this regard. In 2021 Bluebird Bio announced it would withdraw from EU markets due to EU member states’ refusal to make large upfront payments for gene therapies. This was seen as having wider implications: “If there is ultimately no market in Europe (…), will the lack of commercial success be a hindrance to the long-term success of the field more broadly?” (*The Boston Globe* 17/08/2021) Commentators noted the situation was “awkward for Bluebird, showing business priorities don't always align with the biotech's “patients first" mantra” (*The Boston Globe* 14/12/2021).

In one of the most critical pieces in our data set, entitled “Sequencing the human genome hasn’t led to breakthrough”, an American physician further debunked the hope of universal adoption of gene therapies.

In 2000, Francis Collins, then head of the HGP at the National Institutes of Health, predicted, “Perhaps in another 15 or 20 years, you will see a complete transformation in therapeutic medicine.” It is now 2020 and no one carries a genome card. Physicians typically do not examine your DNA to diagnose or treat you, as common serious diseases are rarely caused by single-gene mutations, they cannot be cured by replacing the mutated gene with a normal copy, the premise for gene therapy. Gene therapy has gradually progressed in research along a very bumpy path, which has included accidentally causing leukemia and at least one death, but doctors recently have been successful treating some rare diseases in which a single-gene mutation has had a large effect. Gene therapy for rare single-gene disorders is likely to succeed, but must be tailored to each individual condition. The enormous cost and the relatively small number of patients who can be helped by such a treatment may create insurmountable financial barriers in these cases. For many diseases, gene therapy may never be useful. (*The Boston Globe* 16/02/2020)

The position of patients is particularly difficult in this area. One high-profile story followed two families, each with two girls suffering from sickle cell disease. While one of these families was able to access clinical trials, the girls from the another one were still on the waiting list. As veteran reporter Gina Kolata recounted:

[I]n one family, both girls were freed from their symptoms and are now living normal lives. In the other, the sisters are still suffering and yearning for the chance to rid themselves of the disease. (…) I am haunted by the disparities. (*New York Times* 14/09/2021).

This showed the uneven realities of access to gene therapies, especially for Black Americans. Other families also faced uphill battles to get access to gene therapies. In these stories, parents often featured as petitioners asking for their sick children to be granted a chance to live longer lives. One such case was a crowd-funded access campaign to *Zolgensma* that was closely followed by multiple news outlets in the UK. The parents explained:

“1.7 million is a lot of money but it is a one-off treatment and research has shown that children who have been given it are surviving and thriving five years on,” says Megan [Edward’s mother]. “We would love to see Edward walk and talk and this offers the best chance. Wouldn't all parents want the same?” (*Daily Mail* 15/12/2020).

The large-scale campaign mounted by Edward’s parents was eventually successful. With Zolgensma’s later approval in the UK, the following year their son received the treatment as part of NHS (publicly funded) healthcare.

Yet even successful treatments can carry high economic and emotional burdens. In a personal account that appeared in *The New York Times*, American author and TV host Annabelle Gurwitch described her experience of undergoing a daily targeted therapy regimen for cancer:

My health has stabilized and my oncologist tells me this portends well for long-term management. The medication I take every day retails at $500 a dose. […] Others in my cancer support group with lesser plans report paying between $1,000 and $3,000 a month out of pocket. Last week, I called Becca, a social worker and cancer counsellor. “'I'm so grateful the therapy promises a longer life span,”' I told her, “'but am I your only client who wonders if it wouldn't be better to check out earlier? I don't want to become a burden to my family.”' “'No,” she sighed. “I hear this all day.” […] (*The New York Times* 15/11/2020; despite stating that this statement relates to gene therapy, it appears to relate to a targeted molecular treatment)

As costs for gene therapies are increasing, and new treatments commanding ever-higher payments, this issue is only likely to become more pervasive and pressing. In this regard, patients’ experiences can offer striking contrasts to the more enthusiastic coverage of scientific progress. Our sample showed that expectations of universality and accessibility of gene therapies were questioned both in terms of their economic viability and in terms of real-life barriers to access to treatments – casting doubt on the large-scale adoption of gene therapies for complex conditions.

## VI Discussion

What is striking about the gene therapy imaginary over many decades is its persistent optimism and hope in terms of its clinical potential, despite major setbacks. This resilience is also evident on the level of individual expectations in our sample. Here, while some promises regarding gene therapy are becoming harder to maintain, others continue virtually unchanged, despite evidence that runs counter to their content. The promise of safety and effectiveness of treatments, for instance, has not been drawn into serious question, even when trials failed and individual patients had adverse results – including a widely publicised death. None of these events affected the primary narrative that gene therapies are effective, even after one dose – and safe to use. The promise regarding profitability had at least one widely publicised high-profile case that directly disproves its underlying assumptions – other recent examples are the withdrawal of Glybera from the European market (it had only found one paying patient; Senior 2017), and Pfizer’s withdrawal of its hemophilia gene therapy Beqvez (National Bleeding Disorders Foundation 2025) – but expectations of vast profit clearly still exert a powerful influence. Lastly, the promise of accessibility has seen the most critical and varied coverage, including a range of different perspectives. Patients and professionals (for instance physicians and insurers) are providing accounts based on real-life experience, highlighting issues in gaining access to existing gene therapies. Most strikingly, these issues are not discussed in pieces that primarily focus on safety, effectiveness, or profitability of gene therapies. Here, accessibility remains an undiscussed and optimistic assumption.

But patient stories are not always critical of gene therapies, in fact, they report successful treatments in often glowing terms. As stated at the outset, our analysis deliberately focused on more critical, cross-cutting coverage instead of repeating these straightforwardly enthusiastic pieces. These positive accounts however reiterate the original promise of safety and effectiveness of gene therapies, and show the surprising resilience of this expectation. This can be seen as part of the constant (re)making of a broader imaginary of a revolutionary personalised and genetic or genomic medicine, despite relatively slow progress overall. Some more critical patients’ and physicians’ accounts can give an idea of the complex situation regarding the effectiveness of these new treatments. As most of these treatments have not been around for much more than 5 years, long-term implications are still entirely unknown at present. Some treatments seem to be effective in the long term, while others apparently ‘wear off’. The situation even with existing ‘miracle’ treatments is thus far from clear-cut, as one American paediatrician points out: “I have some kids who are running around like little maniacs. (…) And I have some kids who still have some problems.” The use of these therapies may be life-changing, but not necessarily “disease ending”. Some children who receive them “still require significant support and never regain or develop certain capabilities” (*The Boston Globe* 01/06/2021). This shows the limits of assumptions of one-time curative interventions – which are however not widely discussed in other reports.

This tension between continually optimistic expectations and real-life complexity shows that there is currently an uneasy coexistence of a regime of hope and an emerging regime of truth (after Moreira and Palladino 2005) in the field of gene therapy. We argue that this coexistence is part of a process of actualisation that takes place as a field of anticipated technological and medical change becomes a real-life regime of experience. Brown and Michael analysed a similar moment of re-evaluation in the case of xenotransplantation, a once much anticipated medical innovation using “nonhuman tissue and organs in human transplant surgery” (2003, 5). This medical field has however not proven as successful as originally anticipated, fraught with persistent issues around tissue rejection and transspecies disease contagion. In contrast, gene therapy is much more successful, with actual treatments becoming available at present. Our focus is thus on a regime that did not undergo “significant changes” (Brown and Michael 2003, 8), in fact, it is striking in how far it does not seem to change at all, even in the face of failures and set-backs. This could be connected to the fact that gene therapy is “an innovation concept [that] has a longer history”, as Brown and Michael suspected (2003, 4) – its established nature could render it more resilient against disappointment. Yet objectively there are several at present virtually insurmountable issues, making the future of gene therapy appear increasingly bleak – but it simply does not do so in media accounts.

Some of this optimistic reporting can also reveal the power of media narratives in directing attention. Here, a particular type of story succeeds more readily than others, as reflected in our data set’s overwhelmingly positive slant. Miracle stories are central to each promise, including safety and effectiveness; profitability; and accessibility. However, as the above has shown, increasing reference to real-life experiences of stakeholders in the field can undermine these apparent successes, especially when discussing more than one promise. For instance, the involvement of patients leads to higher complexity both in debates around accessibility, and also in the less patient-centric debate around profitability. These more complex accounts of gene therapy’s promises are however still few and far between, especially within our popular media sample. In contrast to that, Brown and Michael’s analysis consulted stakeholder opinions over time, yielding a more sophisticated and detailed re-assessment of expectations. We can thus see differences in the dynamics of expectations over time in these very distinctive “communities of promise” (Martin, Brown and Kraft 2008) – one undergoes a rapid process of actualisation and reflection in the light of real-life experience, whereas the other maintains a generalised hope, both by design and also by desire.

## VII Conclusion

Disappointments and real-life concerns have, so far, had apparently very little impact on the original and continuing promises of gene therapy. The announcement of a scientific paradigm shift towards the human genome in 2000 clearly marked and contributed to the emergence of a very powerful imaginary, amplified by an occasion in which research suddenly took centre stage even within the global political sphere of the White House. This momentous event epitomised the public race towards the development of popular genetic medicine, and represented a unique entanglement of research, biotech industry, and the public sector. Clearly and deliberately evoking the race for the moon, the moment of (preliminary) completion demonstrated both the mastery of science, and also the centrality of US industry’s role in this new sector. In this context, the availability of gene therapies over twenty years on clearly tells a story of success, as a number of the original participants of this moment also stated. The eventual success of their agenda is however still far from assured, as the more critical voices above pointed out. The path to a true “[t]wenty-first century medicine” (Verma and Weitzman 2005) is still a long and complex one.

But from its inception, this genetic medicine has captured the imagination of many, both those closely involved in its creation and also those of the general public. This still shows in the data under consideration here, which indicates almost a double-life of the concept of gene therapy. On the one hand, the promise of this revolutionary treatment holds sway, almost without change – while on the other, some personal experiences of gene therapy can show the flip side of this new treatment regime. Unaddressed structural challenges are also becoming increasingly difficult to ignore, especially as gene therapies are commanding higher and higher premiums. Clearly there is work to be done until this medicine can fulfil its promise, or, more accurately, a real-life version of this promise.
